# Catalytic deAMPylation in AMPylation-inhibitory/assistant forms of FICD protein

**DOI:** 10.3389/fchem.2023.1077188

**Published:** 2023-01-25

**Authors:** Meili Liu, Li Li, Zhiqin Wang, Shuang Wang, Xiaowen Tang

**Affiliations:** ^1^ Department of Medical Chemistry, School of Pharmacy, Qingdao University, Qingdao, China; ^2^ Department of Civil and Architectural Engineering, University of Miami, Coral Gables, FL, United States; ^3^ Department of Stomatology, Huangdao District Central Hospital, Qingdao, China

**Keywords:** FICD protein, deAMPylation, inhibitory helix, QM/MM MD, catalytic mechanism

## Abstract

DeAMPylation, as a reversible reaction of AMPylation and mediated by the endoplasmic reticulum-localized enzyme FICD (filamentation induced by cAMP domain protein, also known as HYPE), is an important process in protein posttranslational modifications (PTMs). Elucidating the function and catalytic details of FICD is of vital importance to provide a comprehensive understanding of protein folding homeostasis. However, the detailed deAMPylation mechanism is still unclear. Furthermore, the role of a conserved glutamine (Glu234), that plays an inhibitory role in the AMPylation response, is still an open question in the deAMPylation process. In the present work, the elaborated deAMPylation mechanisms with AMPylation-inhibitory/assistant forms of FICD (wild type and Glu234Ala mutant) were investigated based on the QM(DFT)/MM MD approach. The results revealed that deAMPylation was triggered by proton transfer from protonated histidine (His363) to AMPylated threonine, instead of a nucleophilic attack of water molecules adding to the phosphorus of AMP. The free energy barrier of deAMPylation in the wild type (∼17.3 kcal/mol) is consistent with that in the Glu234Ala mutant of FICD (∼17.1 kcal/mol), suggesting that the alteration of the Glu234 residue does not affect the deAMPylation reaction and indirectly verifying the inducement of deAMPylation in FICD. In the wild type, the proton in the nucleophilic water molecule is transferred to Glu234, whereas it is delivered to Asp367 through the hydrogen-bond network of coordinated water molecules in the Glu234Ala mutant. The present findings were inspirational for understanding the catalytic and inhibitory mechanisms of FICD-mediated AMP transfer, paving the way for further studies on the physiological role of FICD protein.

## Graphical Abstract

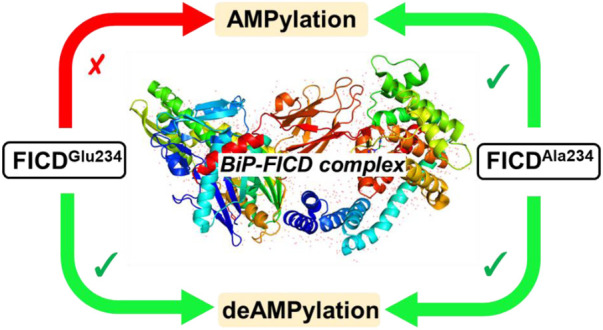

## 1 Introduction

Posttranslational modifications (PTMs) of proteins are a regulatory mechanism that enables molecules to control and diversify cell functions. Misregulation is often associated with severe pathology, including autoimmune diseases and cancer (Brown et al., 1971; O'Shea et al., 2013). Conserved from bacteria to humans, protein phosphorylation, acetylation, and methylation are almost ubiquitous posttranslational mechanisms used to control and regulate complex signaling processes and have been explored extensively (Ham et al., 2014; Preissler et al., 2015; Sanyal et al., 2015; Preissler et al., 2017; Casey et al., 2018). Protein AMPylation as a novel regulatory mechanism that could mediate eukaryotic signaling processes has joined this list in recent years. Similar to AMPylation, deAMPylation as a reversible process is a potential regulatory mechanism that could mediate eukaryotic signaling processes.

**SCHEME 1:**
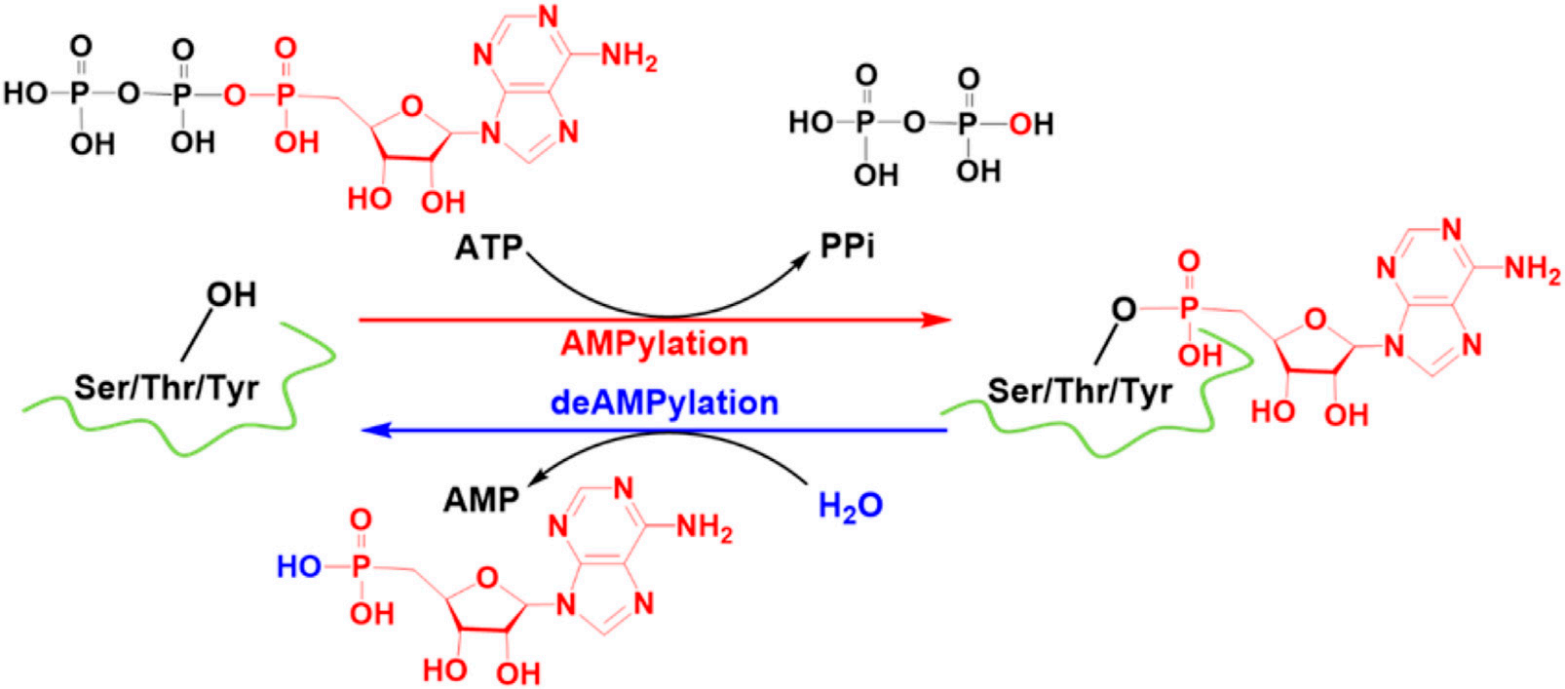
FICD-catalyzed AMPylation and deAMPylation processes. Some crucial atoms/groups are labeled by color.

Little is known about the AMPylation in protein regulation, and less is known about deAMPylation of proteins. Nonetheless, among the limited studies, structural insights into the mechanism (Preissler et al., 2017; Luong et al., 2010) and mechanistic studies at the atomic level (Liu et al., 2021) toward AMPylation have been disclosed. In contrast, only a few proteins with deAMPylating activity have been identified. The two known bacterial proteins with deAMPylating activity are SidD from the human pathogen *Legionella pneumophila* (Chen et al., 2013) and bifunctional GS-ATase from *Escherichia coli* (Anderson et al., 1970). The first conserved eukaryotic AMPylator filamentation induced by cAMP domain (FICD) was identified by Ron and co-workers (Preissler et al., 2017). FICD, a single bifunctional enzyme, belongs to a family of bacterial FIC domain proteins, which is responsible for both AMPylation (Ham et al., 2014; Preissler et al., 2015; Sanyal et al., 2015) and deAMPylation (Casey et al., 2017; Preissler et al., 2017; Veyron et al., 2019) of endoplasmic reticulum (ER) Hsp70. BiP is a key component of the unfolded protein response (UPR), which is a major pathway whereby cells respond to ER stress (Zhao et al., 2006). The AMPylation of BiP, which refers to covalent attachment of an ATP-derived AMP moiety to the Thr518 hydroxyl group, is called the best-defined BiP PTMs. It is well-known that AMPylation of BiP is triggered by reduction of ER stress; however, the mechanism for the subsequent deAMPylation process under UPR induction is still unknown. DeAMPylation is always considered a reversible step of AMPylation due to the two processes being catalyzed by the same conserved Fic domain. Actually, deAMPylation is not the exact reverse of AMPylation, as deAMPylation of BiP leads to release of AMP rather than ATP production (Scheme 1). Thus, FICD is capable of catalyzing two distinct reactions: AMPylation and deAMPylation. Additionally, viewing the structures of two bacterial deAMPylation enzymes, a metal-dependent protein phosphatase folding pattern (Chen et al., 2013) or nucleotidyl transferase folding pattern (Xu et al., 2004; Xu et al., 2010), is utilized to catalyze the binuclear Mg^2+^-facilitated deAMPylation with a hydrolytic (Chen et al., 2013) or phosphorolytic (Anderson et al., 1970) nature. However, the mammalian AMPylated BiP–FICD complex contains a single divalent cation binding site, which is evolutionarily and structurally divergent from bacterial deAMPylases, and likely catalyzes deAMPylation with a distinct mechanism.

Interestingly, the AMPylation process is often auto-inhibited by a glutamate-containing alpha helix (α_inh_) (Engel et al., 2012; Goepfert et al., 2013). Of specific note, Glu234, recognized as an AMPylation inhibitor, is considered an essential factor for deAMPylation in FICD (Preissler et al., 2017). Furthermore, a recent investigation reveals that deAMPylation activity of the *Enterococcus faecalis* Fic protein (EfFic) is dependent on a glutamate homologous to Glu234 in FICD (Veyron et al., 2019), suggesting the conservation of the catalytic mechanism among Fic enzymes. However, whether Glu234 is irreplaceable for deAMPylation is still uncertain, and the role of Glu234 in the oligomeric state-dependent regulation of FICD’s mutually antagonistic activities remains incompletely understood.

It is most recently that Ron and co-workers (Perera et al., 2021) resolved the crystal structure of the deAMPylation Michaelis complex formed between mammalian AMPylated-BiP and FICD, which made it possible to disclose the reaction details of deAMPylation in mammals at the atomic level. In the present study, the state-of-the-art Born–Oppenheimer quantum mechanical/molecular mechanical molecular dynamics (QM/MM MD) simulation was employed to systematically investigate the deAMPylation mechanism of AMPylated-BiP catalyzed by the FICD enzyme in detail. Proton transfer from protonated histidine (His363) in FICD to AMPylated threonine (Thr518) in BiP is demonstrated to initiate the deAMPylation process, instead of the general viewpoint that refers to a nucleophilic attack of water molecules adding to the phosphorus of AMP. Moreover, it is also revealed that the crucial AMPylation-inhibiting Glu234 that has proved to be essential in the bacterial deAMPylation process is alterable in mammals. Our present research sheds more light on the comprehension of the physiological role of FICD protein and PTMs.

## 2 Materials and methods

### 2.1 Model preparation

The initial deAMPylation models were constructed based on the monomeric FICD and AMPylated BiP complex from the RCSB PDB (PDB code: 7B7Z) (Perera et al., 2021). When constructing the wild type deAMPylation research model, the AMPylated Thr518 residue and coordination mode of Mg^2+^ were retained. The engineered mutations in the crystal structure were recovered, and the missing residues and atoms were also complemented. The H++ program was employed to estimate the protonation states of titratable residues (Gordon et al., 2005). Furthermore, the individual local hydrogen bond networks were also carefully examined. Of specific note, His363 in FICD was recognized to be protonated, which inherited the product structure of the preceding AMPylation stage (Liu et al., 2021). Eventually, the constructed deAMPylation model was used for the following classical molecular dynamics (MD) and QM/MM simulations.

### 2.2 Classical molecular dynamics simulation and trajectory analysis

The classical MD simulations were performed using the AMBER18 molecular simulation package (Case et al., 2018). The AMPylated Thr518 was redefined as a non-standard amino acid, for which the force field parameters were generated from the general AMBER force field (GAFF) (Wang et al., 2004), and the partial atomic charges were defined by the restrained electrostatic potential (RESP) charge (Bayly et al., 1993) based on HF/6-31G* calculation with the Gaussian 09 package (Frisch et al., 2009). The reliability of the parameter fitting procedure for the ligand was demonstrated in our previous studies (Liu et al., 2014). Additionally, the general amino acids in FICD and BiP were described using Amber ff14SB force field (Duan et al., 2003), and solvent water molecules were simulated with the TIP3P model (Jorgensen et al., 1983). The cubic water box model with periodic boundary condition was used to create the solvent environment. Eventually, the *tleap* program (Case et al., 2018) in AMBER18 was performed to generate the initial coordinates and topology files of the neutralized and solvated deAMPylation model. Before the final production MD simulation, the routine gradient minimization, programmed heating (from 0 to 310 K under NVT ensemble for 100 ps), and density balance (NPT ensemble for 100 ps at 310 K and 1.0 atm) were carried out. Afterward, 100-ns NVT production MD simulations with a target temperature of 310 K were performed to produce trajectories. During the MD simulations, the SHAKE algorithm (Ryckaert et al., 1977) was applied to constrain the high-frequency stretching vibration of all hydrogen-containing bonds, and a cutoff of 12 Å was set for both van der Waals (LJ-12 potential) and electrostatic interactions (PME strategy). Finally, the last snapshot from the stable MD trajectories was chosen to build the initial model for subsequent QM/MM simulations. More validations on the model reliability (consistency analyses on crystal structure, representative structure of dominant cluster, and selected research model) are provided in Supporting Information.

### 2.3 QM/MM simulation and free energy calculation

The present QM/MM calculations were performed using the interfaced QChem-AMBER12 programs (Shao et al., 2006) Residues that refer to the deAMPylation process directly (AMPylated Thr518 in BiP, Glu234/Ala234 and protonated His363 in FICD, and the nucleophilic water molecule) were considered in the QM region undoubtedly; moreover, Mg^2+^ ions and its coordinated residues (Asp367 in FICD and four water molecules) were also considered owing to the remarkable charge dispersion effects. The remaining atoms were considered in the MM region. An improved pseudo-bond approach (Zhang et al., 1999; Chen et al., 2005; Zhang YK., 2006) was employed to treat the boundary of the two regions. The QM atoms were described with the M06-2X/6-31G(d) (Zhao and Truhlar, 2007; Zhao and Truhlar, 2008) level, which is widely used in investigations of enzymatic reaction (Zhang et al., 2016; Zhang et al., 2018; Tang et al., 2020; Zhang et al., 2020; Zhuang et al., 2022); eventually, 726 and 662 basis functions were contained in the wild type and mutant deAMPylation system, respectively. The MM region was described with the same molecular mechanical force field as in the preceding classical MD simulation. The phosphorus atom at AMP was defined as the center for electrostatic coupling, and a cutoff of 12 Å was set for van der Waals (Lennard–Jones potential function) and electrostatic (dual-focal ai-QM/MM-PME approach) interactions (Zhou et al., 2016).

**FIGURE 1:**
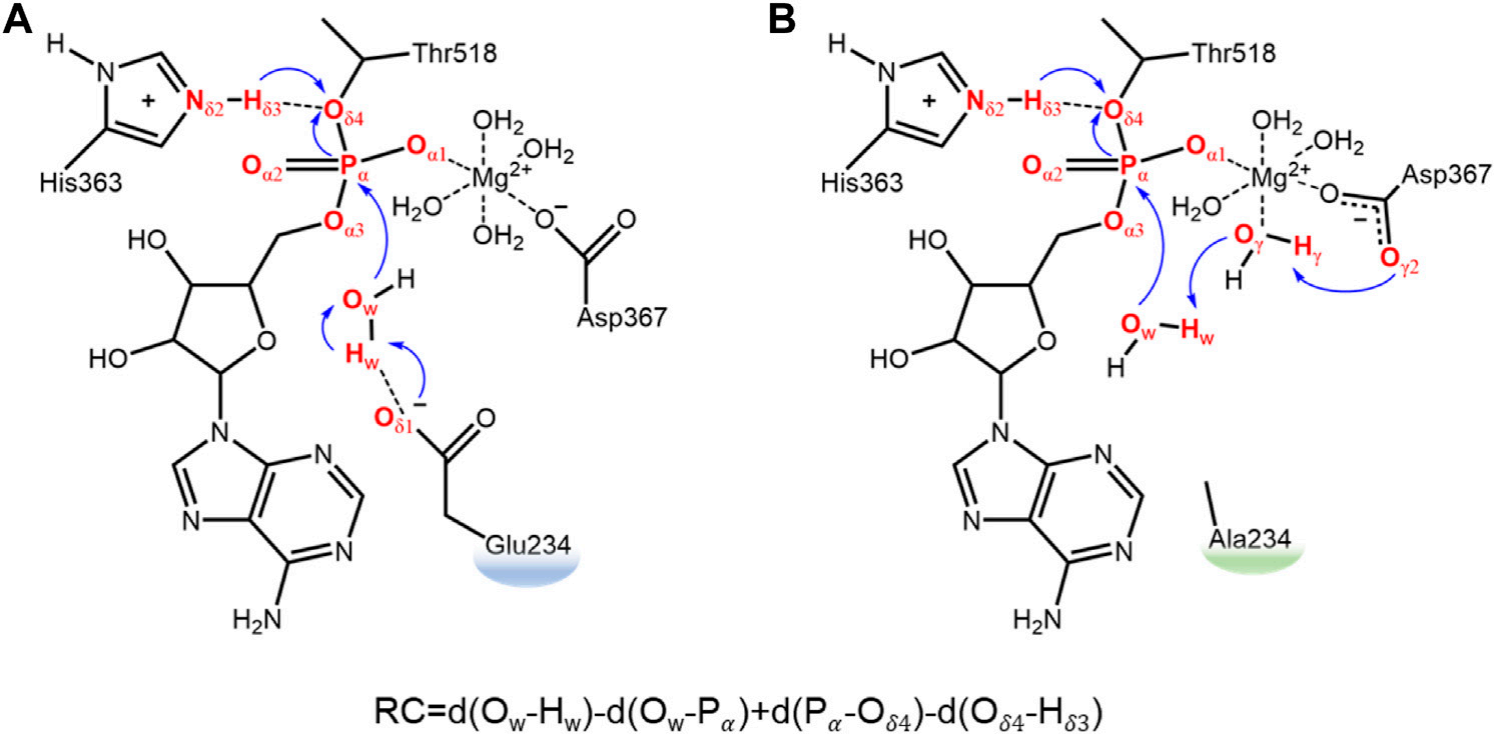
Schematic research model and defined reaction coordinates for the deAMPylation process in the FICD wild type **(A)** and Glu234Ala mutant **(B)**. Electron transfer path of deAMPylation is labeled in blue arrows, and atoms involved directly in this process are colored in red.

The deAMPylation system was relaxed with 5 ps QM/MM MD simulations after a QM/MM minimization for several iterations. The fully relaxed conformation was used to search the minimum energy path with the reaction coordinate (RC) driving method (Zhang et al., 2000) according to the defined reaction coordinates as shown in Figure 1. Afterward, free energy perturbation (FEP) simulation (500 ps) was employed to equilibrate the MM region of the structures in the minimum energy path, in which the QM region was fixed at the corresponding reaction coordinate. The fully equilibrated structures were used for the subsequent biased-potential based QM/MM MD umbrella sampling (Torrie and Valleau, 1977). The Langevin thermostat method (Davidchack et al., 2009) was adopted for temperature control (310 K), and the Beeman algorithm (Beeman D., 1976) was used to integrate the Newton equations of motion. Finally, 19 windows for the wild type and 29 windows for the Glu234Ala mutant deAMPylation system were generated, and each window was simulated for at least 20 ps. The overlaps of sampling between neighboring windows were checked to confirm if each window was adequately sampled along the proper reaction coordinate, and the final free energy profile was calculated with the WHAM program (Kumar et al., 1992; Souaille and Roux, 2001). The convergence of QM/MM MD umbrella sampling was estimated by the free energy profile gap calculated from different time spans (10–20 ps, 10–15 ps, and 15–20 ps). The present simulation and free energy calculation protocols have been successfully utilized and validated in previous studies (Zhang et al., 2016; Zhang et al., 2018; Tang et al., 2020; Zhang et al., 2020; Zhuang et al., 2022).

## 3 Results and discussion

Two deAMPylation systems were constructed in the present work, which were involved in the AMPylated–BiP complexed with the wild type FICD-containing Glu234 residue and a FICD variant with Ala234 residue. Stable research models are usually strictly indispensable to computational simulations. The root-mean-square deviations (RMSDs) of 100-ns MD simulations (Supplementary Figure S4) indicate that the two complexes have reached the thermodynamic stable state and satisfy the needs of the subsequent QM/MM simulations.

### 3.1 Energy and distance changes of deAMPylation

**FIGURE 2:**
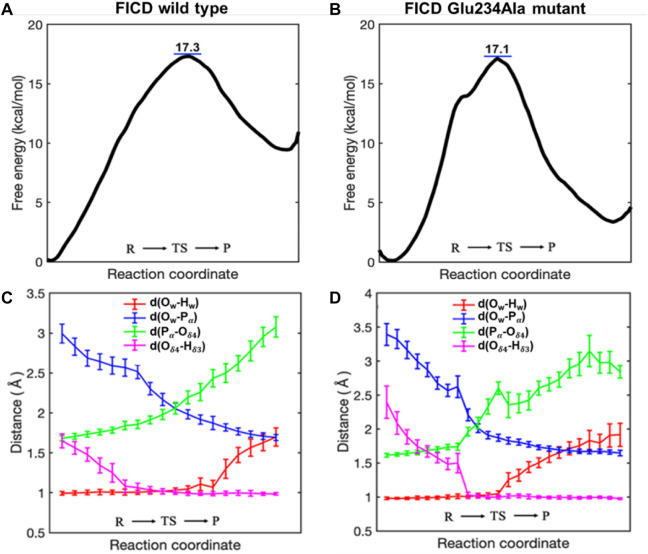
Free energy profiles **(A, B)** and pivotal distance changes **(C, D)** along the reaction coordinate (RC) of the deAMPylation process in the wild type and Glu234Ala mutant system.

As shown in Figure 2A, the calculated free energy barrier for deAMPylation in the wild type is 17.3 kcal/mol, which is consistent with the measured apparent kinetic constant in the experiment (kcat is ∼26 s^−1^, about 16 kcal/mol as converted into an energy barrier) (Luong et al., 2010). Moreover, the barrier in the Glu234Ala mutant system (17.1 kcal/mol shown in Figure 2B) is almost identical with that in the wild type. In addition, the thermal effects of deAMPylation in both systems are endothermic, in spite of the difference in reaction heat (∼10 kcal/mol in the wild type and ∼3 kcal/mol in the Glu234Ala mutant system). The identical kinetic free energy barrier and thermodynamics effect reveal that the two systems possess the equivalent ability of accomplishing the deAMPylation process, that is, the crucial AMPylation-inhibitory Glu234 residue is not conserved in the deAMPylation process.

Variation trends of crucial distances along the wild type and Glu234Ala mutant system (Figure 2C, D) are also consistent, which indicates deAMPylation in the two systems occurs with the same mechanism. During the whole process from the reactant (R) *via* a transition state (TS) to the final product (P), the variation trends of P_α_–O_w_ and P_α_–O_δ4_ suggest dissociation of the AMPylated–BiP complex. The distance between O_w_ and H_w_ is almost unchanged in the first half (from R to TS) and obviously increases in the latter half (from R to TS), whereas the distance between O_δ4_ and H_δ3_ exhibits the opposite behavior, which reveals that the proton transfer from protonated histidine (His363) in FICD to AMPylated threonine (Thr518) in BiP is the initiator of deAMPylation, instead of nucleophilic attack of water molecules adding to the phosphorus of the AMP moiety.

### 3.2 DeAMPylation mechanism for the wild type and Glu234Ala mutants of FICD

**FIGURE 3:**
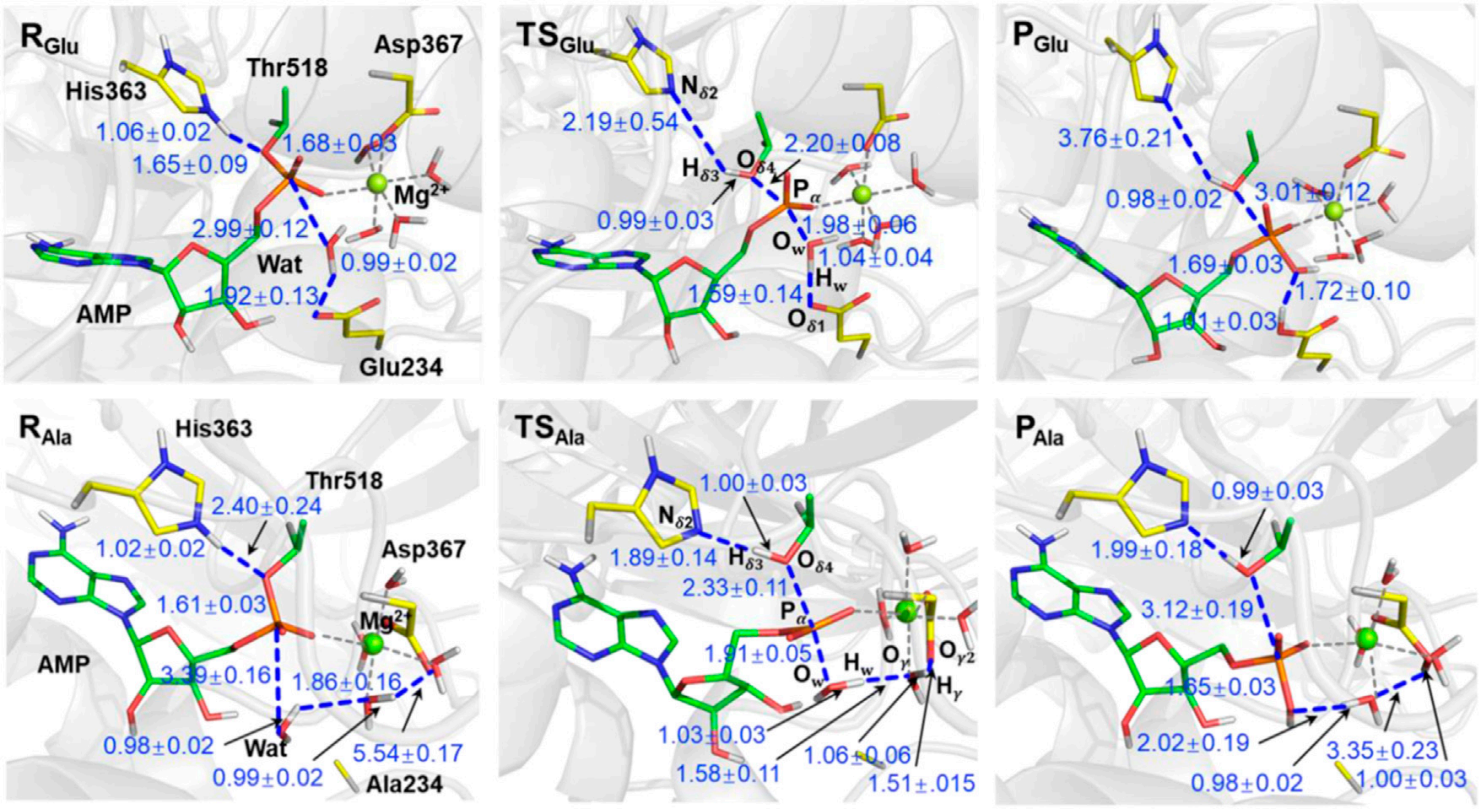
Representative structures of reactants (R), transition states (TS), and products (P) identified according to the free energy profile of Figure 2. Structures in the wild type and Glu234Ala mutant system are distinguished by the subscript (Glu and Ala). Atoms are colored for clarity, C (yellow for residues and green for AMP), P (orange), O (red), N (blue), and H (white). Distances are given in angstrom.

Going from the reactant to the product in the wild type system as shown in Figure 3, the proton (H_δ3_) in the protonated histidine (His363) residue is transferred to oxygen in the AMP-Thr518 group (O_δ4_), activating the deAMPylation reaction with length of a bond pair N_δ2_–H_δ3_/H_δ3_–O_δ4_ changing from ∼1.06/∼1.65 Å to ∼3.76/∼0.98 Å. At the same time, the tetrahedral configuration in R_Glu_ composed of the AMP group and Thr518 is transformed into a PO_3_ planar triangle with a leaving tendency of the Thr518 residue (P_α_-O_δ4_ of ∼2.20 Å) and an approaching tendency of nucleophilic water P_α_-O_w_ of ∼1.98 Å) in the transition state (TS_Glu_). It is worth noting that O_δ4_, O_w_, and PO_3_ in the AMP group form a standard triangular bipyramid (corresponding dihedral angles are shown in Supplementary Figure S5). It is adequately prepared for the nucleophilic attack from the tetrahedral configuration composed of the AMP group and Thr518 to PO_3_ planar triangle. During the whole process, the P_α_–O_δ4_ bond breaks with the bond length changing from ∼1.68 Å to ∼3.01 Å, and a new tetrahedral configuration composed of AMP and hydroxide from the nucleophilic water molecule was generated, suggesting the achievement of deAMPylation. Additionally, in product (P_Glu_), the nucleophilic water transfers a proton to Glu234 to form a H_w_–O_δ4_ bond (∼1.01 Å) and attacks P_α_ atom to form a P_α_-O_w_ bond (∼1.69 Å), and there is still remarkable hydrogen bond interactions between the two segments of the dissociated water molecule, for which the distance of O_w_ and H_w_ is ∼1.72 Å. The variation for some crucial distances involved in the reaction coordinate of the deAMPylation reaction is shown in Supplementary Figure S6.

For the Glu234Ala mutant of FICD complexed with AMPylated–BiP, the divalent metal Mg^2+^ ion maintains hexacoordination with the Asp367 residue, AMP-O_α_, and four water molecules. Focused on the reactant (R_Ala_), the nucleophilic water molecule, that has a hydrogen bond interaction with Glu234 in the wild type, establishes strong interactions (H_w_-O_γ_, ∼1.86 Å) with coordinated water molecules from the divalent metal center. At the same time, no interactions were observed between the coordinated water molecule and Asp367 residue. In the beginning of the deAMPylation reaction in the Glu234Ala system, the first stage is also His363 transferring a proton to the AMPylated-Thr518 group, which is in accordance with that in the wild type system. Subsequently, the planar triangle configuration of PO_3_ in TS_Ala_ is also modified from the tetrahedral configuration of the AMP group and Thr518 in R_Ala_. Particularly, P_α_-O_δ4_ bond and P_α_-O_w_ bond are ∼2.33 Å and ∼1.91 Å in TS_Ala_, respectively, also giving a triangular bipyramid configuration. The subtle differences in transition states (TS_Glu_ and TS_Ala_) of the two systems indicate the same mechanism for proton transformation between His363 and AMPylated-Thr518 (the first half of deAMPylation). Distinctive processes happened in the latter half of deAMPylation in the Glu234Ala system, and a long-chain proton transfer path from O_w_ *via* O_γ_ to O_γ2_ (atom labels in Figure 1) in the dissociation of the nucleophilic water molecule is observed, in which a series of distances referring to a string of atoms O_w_-H_w_-O_γ_-H_γ_-O_γ2_ change from the initial R_Ala_ (∼0.98 Å, ∼1.86 Å, ∼0.99 Å, and ∼5.54 Å) to the final P_Ala_ (∼2.02 Å, ∼0.98 Å, ∼3.35 Å, and ∼1.00 Å) (Figure 3). In addition, the hydroxide, a component of the trigonal bipyramid in TS_Ala_, is stabilized by the long-chain hydrogen network interactions, differing with that only interact with Glu234 in TS_Glu_ of the wild type system. For the nucleophilic water molecule in P_Ala_, it was dissociated with the hydroxide segment covalently bonding to phosphorus (O_w_-P_*α*_ of ∼1.65 Å) and the other segment (proton, H_w_) captured by a coordinated water molecule (H_w_-O_γ_ of ∼0.98 Å). Similar to P_Glu_, there is also a remarkable hydrogen bond interaction between the hydroxide and proton segment of nucleophilic water molecule, for which the distance between O_w_ and H_w_ is ∼2.02 Å. The variations for the crucial angles (plane and dihedral) and distances involved in the reaction coordinate of deAMPylated for the FICD complex BiP in the Glu234Ala mutant system are shown in Supporting Information (Supplementary Figures S5–S7).

Furthermore, whether the deAMPylation mechanism applied in the Glu234Ala mutant system can also occur in the wild type system was also evaluated (Supplementary Figure S8). The scanned potential energy surface indicated that the mechanism adopted in the Glu234Ala mutant is not applicable in the wild type system. A plausible explanation is that the polarization effect of the nucleophilic water molecule directly polarized by Glu234 is much stronger than that through a series of proton transfer mechanisms (the mechanism in the Glu234Ala mutant system). Therefore, the nucleophilic water molecule cannot be polarized by Mg-coordinated H_2_O or Asp367 through a series of proton transfer mechanisms to participate in the deAMPylation reaction, owing to the existence of Glu234.

### 3.3 Differences of deAMPylation in wild type and Glu234Ala mutants

**FIGURE 4:**
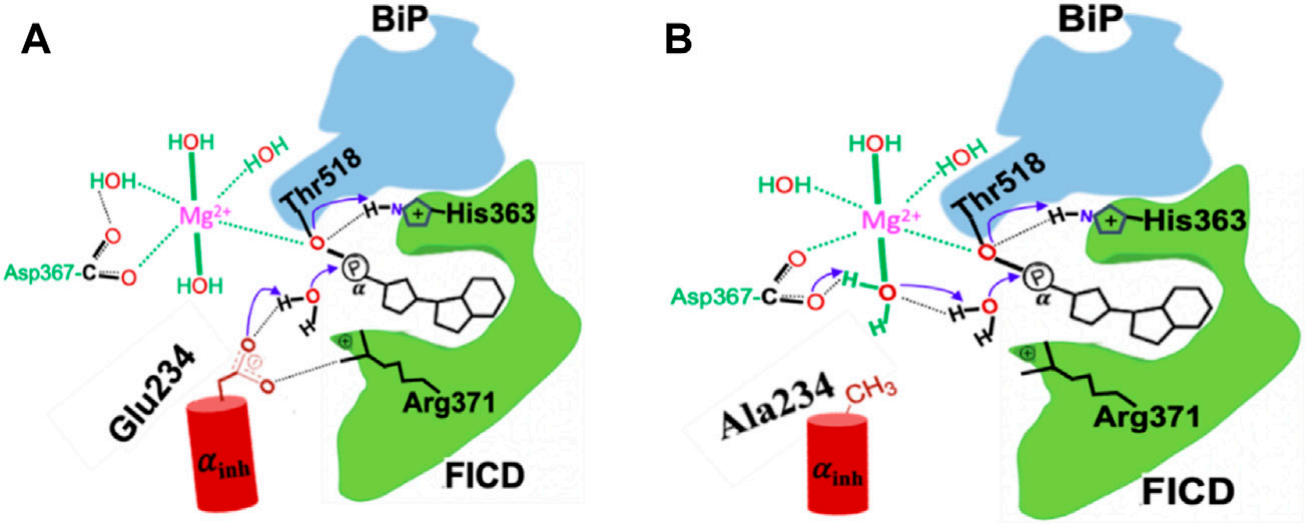
Overall deAMPylation mechanism with FICD in the wild type **(A)** and Glu234Ala mutants **(B)**.

In the present work, the catalytic mechanism of deAMPylation in FICD is investigated in wild type and Glu234Ala mutants. The free energy barrier is 17.3 kcal/mol in the wild type and 17.1 kcal/mol in the Glu234Ala mutant (Figure 2). The results reveal that, with either Glu234 residue or Ala234 residue, FICD complexed with AMPylated–BiP can achieve the catalytic deAMPylation reaction through the same mechanism with different dissociative ways of the nucleophilic water molecule (Figure 3; Figure 4). In two models, the positive charge provided by Mg^2+^ increases the electrophilicity of the phosphorus and stabilizes the negative charge of the intermediate. Configuration of the pentavalent phosphorus intermediate is rearranged and the phosphorester bond is cleaved, as elicited by the capture of a proton provided by the conserved glutamate in the wild type or the long proton chain in the Glu234Ala mutant. Furthermore, geometric analysis illustrates that the angle of O_δ4_-P_α_-O_w_ involved in the nucleophilic attack in two models is over 160° during the deAMPylation process (Supplementary Figure S7), indicating the feasibility of the substitution reaction. For the two models, the hydrolytic deAMPylation in the wild type and Glu234Ala mutants is triggered by proton transfer from protonated histidine (His363) to the oxygen atom of AMPylated threonine (O_δ4_ in Thr518), instead of a generally nucleophilic attack of water molecules adding to the phosphorus of the AMP moiety. Additionally, there are still some differences on the reaction details of the two models as displayed in Figure 2. It is worth noting that the reaction heats are inconsistent in the two reaction systems (∼10 kcal/mol in wild type vs. ∼3 kcal/mol in the Glu234Ala mutant). A reasonable explanation could be focused on the different charge dispersion modes of the dissociated nucleophilic water molecule in the two systems. Only a hydrogen bond interaction (O_w_-H_w_, ∼1.72 Å) refers to Glu234 is provided in the wild type system; in comparison, a series of hydrogen bond networks refer to the coordinated water and the solvent environment can be provided in the Glu234Ala mutant system. Undoubtedly, the latter pattern exhibits more sufficient charge dispersion effect, giving a more stable product structure than the former pattern.

### 3.4 Tolerance to AMPylation-inhibitory/assistant forms of FICD protein

Notably, deAMPylation and AMPylation are the two processes of a reversible reaction. To further understand the reversible processes of deAMPylation and AMPylation, the AMPylation reaction in our former work and deAMPylation in our current work including wild type and Glu234Ala mutant systems were further analyzed (Supplementary Table S1). The energy barrier of AMPylation with wild type FICD (the motif with Glu234 was defined as an inhibitory α-helix) is 38.7 kcal/mol (Liu et al., 2021), while the corresponding energy barrier of the deAMPylation reaction is 17.3 kcal/mol. So far, experimental observation (Bunney et al., 2014) and theoretical work (Liu et al., 2021) have shown a key inhibitory role of inhibitory glutamate Glu234 on the inhibitory helix (α_inh_) for the AMPylation reaction. For deAMPylation, Glu234 acts as a proton acceptor in the deAMPylation reaction, rather than playing the role of activating the water molecule. Meanwhile, it also can stabilize the nucleophilic water molecule and promote of proton transfer. Interestingly, when Glu234 is mutated as Ala234, the energy barriers of AMPylation and deAMPylation were 14.7 kcal/mol and 17.1 kcal/mol, respectively, revealing that the Ala234 residue is helpful to AMPylation and does not interfere with the deAMPylation. It very well explains why the mutation of Glu234 to Ala234 is not performed in the crystal structure elucidation experiments for the deAMPylation reaction (Perera et al., 2021). This also helps us understand the regulating mechanism of AMPylation and deAMPylation *in vivo* by controlling the endoplasmic reticulum (ER) pressure.

## 4 Conclusion

In the present computational study, the hydrolytic deAMPylation in the FICD complexed with AMPylated–BiP is studied using a more recent crystallographic structure with AMPylated–Thr518 residue at the active site, which allows us to study the deAMPylation mechanism at the atomic level. Two reaction systems, AMPylation-inhibitory (wild type) and AMPylation-assistant (Glu234Ala variant), are constructed for the theoretical simulations. The results suggest that His363 acts as a catalytic acid to protonate the phosphoryl group of AMPylated–BiP and further triggers the deAMPylation process. The free energy barriers are estimated to be ∼17 kcal/mol for both of the two systems, indicating the amino acid site of Glu234 is pivotal to the AMPylation process and insensitive to the subsequent deAMPylation process. The present work served as the first theoretical evidence for the deAMPylation reaction of the prevalent PTMs. It not only provides mechanical and structural details for the deAMPylation reaction but also paves the way for further studies on the physiological role of the FICD protein.

## Data Availability

The original contributions presented in the study are included in the article/Supplementary Material; further inquiries can be directed to the corresponding authors.
