# Supplementary material for: Catalytic deAMPylation in AMPylation-inhibitory/assistant forms of FICD protein
Source: Front Chem. 2023 Jan 25;11:1077188. doi: 10.3389/fchem.2023.1077188 (PMC9905249; doi:10.3389/fchem.2023.1077188)
Supplement: Supplementary file 1 [file DataSheet1.docx]

**Supporting Information**

**Catalytic deAMPylation** **in AMPylation-inhibitory/assistant forms of FICD protein**

Meili Liu^1,2^, Li Li^1^, Zhiqin Wang^1^, Shuang Wang^1,3,^*, Xiaowen Tang^1,^*

^1^ Department of Medical Chemistry, School of Pharmacy, Qingdao University, Qingdao, China

^2^ Department of Civil and Architectural Engineering, University of Miami, Coral Gables, FL, USA

^3^ Department of Stomatology, Huangdao District Central Hospital, Qingdao, China

* To whom correspondence should be addressed, E-mail: [ws1201youyou@163.com](mailto:ws1201youyou@163.com) (S. Wang) and [xwtang1219@qdu.edu.cn](mailto:xwtang1219@qdu.edu.cn) (X. Tang).

**Contents**

Validations on the model reliability; consistency analysis of crystal and MD models (Figure S1), comparison of selected QM/MM model with the most representative structure in the dominant cluster for the MD trajectory of wild type model (Figure S2) and Glu234Ala mutant model (Figure S3); root-mean-square deviations along MD simulations (Figure S4); variations of dihedral angles (Figure S5), crucial distances (Figure S6), plane angles (Figure S7) along the deAMPylation process; evaluation of an alternative deAMPylation mechanism in wild type (Figure S8); crucial information for AMPyaltion and deAMPyaltion (Table S1); and predicted pKa values for titratable residues (Table S2).

**Validations on the model reliability**

It is usually essential to perform molecular dynamics (MD) simulations when investigating enzyme catalyzed reaction mechanism with QM/MM MD approach. The Michaelis complexes obtained from protein database are usually engineered mutants (such as the structure used in the present work) or incomplete (missing amino acid side chains and even flexible loops). Therefore, some additional modifications (adding hydrogens, deleting some non-standard groups, fixing the mutated/missing residues) are implemented to obtain a more reasonable reaction model. Therefore, MD simulations are required to relax the modified structure and finally give the thermodynamics equilibrated model. An overlap of the crystal and MD structure are shown in Figure S1. The tertiary structures of the two models are almost identical with overall RMSD less than 2.1 Å. Notably, protein structures around the reaction site can be overlapped exactly and conformations of some crucial resides refers to the deAMPylation process are also consistent. In consequence, the reaction model from MD simulation is in accordance with the crystal structure.

For evaluating the reliability of the selected QM/MM models, cluster analyses for the MD trajectory of wild type and Glu234Ala mutant model were performed, and comparison of selected QM/MM model and the most representative structure in the dominant cluster was also discussed. As displayed in Figure S2-S3, the dominant cluster (Cluster 1, proportion is over 50%) has been found in MD trajectories cluster analysis of both wild type and Glu234Ala mutant model, and the selected QM/MM model is classified into the dominant cluster. The overall structure overlap indicates that the selected QM/MM model is consistent with the most representative structure of Cluster 1 (RMSD≈0.30 Å). Moreover, some key residues and distances in catalytic site are also consistent. In sum, the models adopted for QM/MM simulation were reliable.


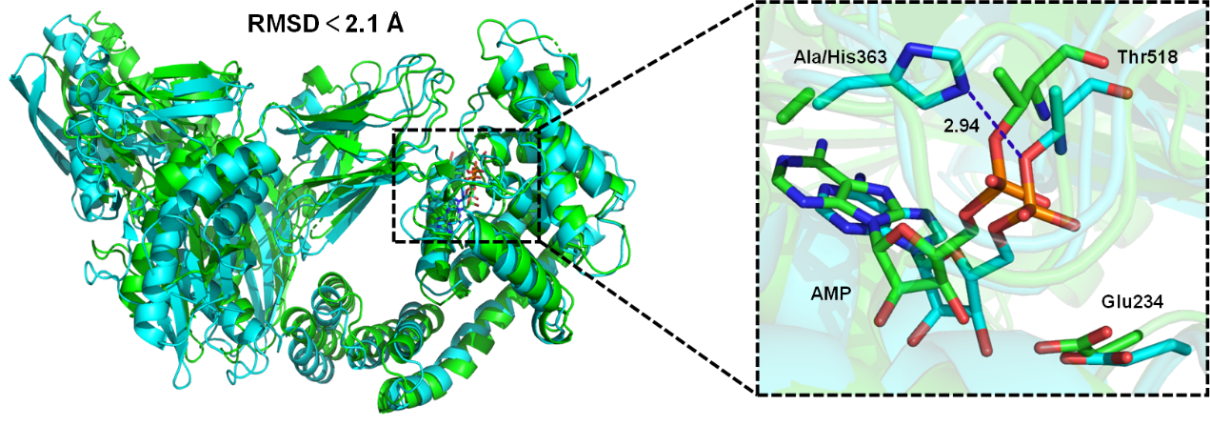


**Figure S1.** Overlap of overall structure and key residues in catalytic site of crystal and MD models. The overall model structures were shown in ribbon form and key residues in catalytic site were displayed with ball and sticks model. The crystal structure was labeled in green and the MD structure was labeled in cyan. The distance that refers to the triggering of deAMPylation (N_δ2_-O_δ4_) was shown with unit of Å.


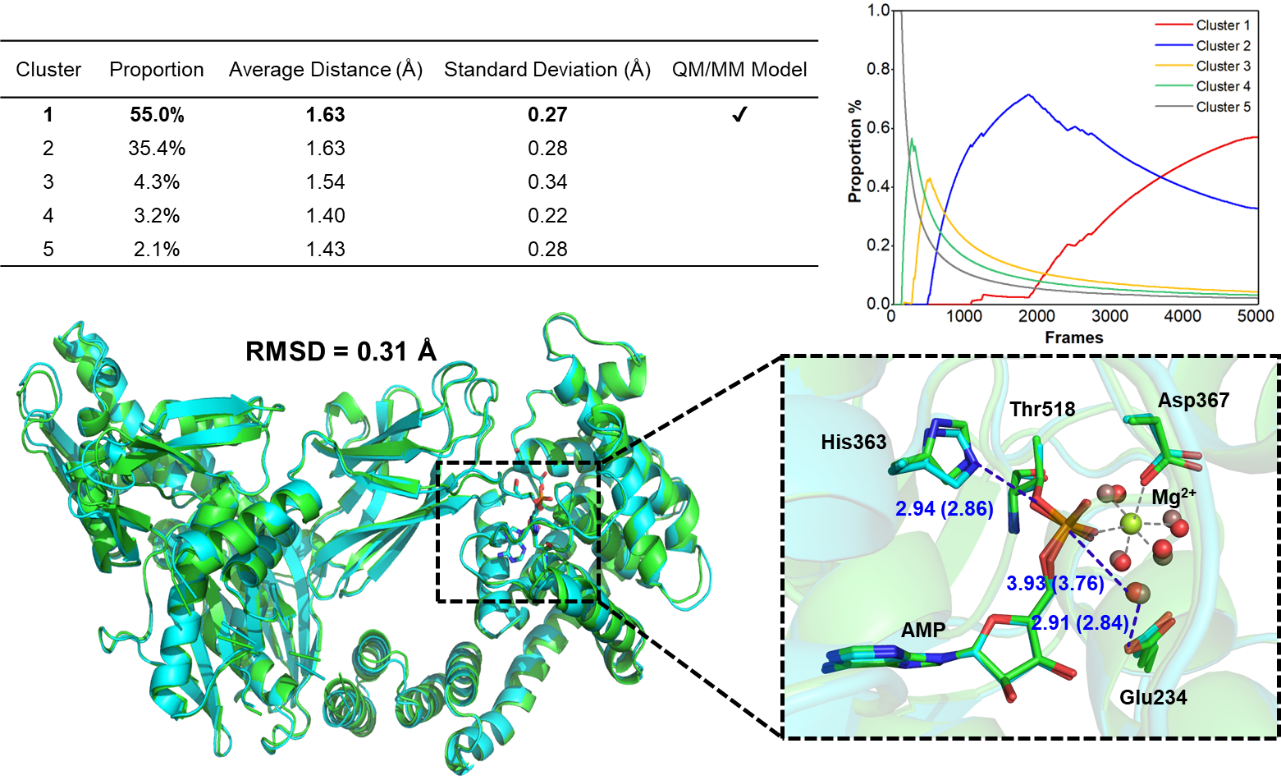


**Figure S2.** Cluster analysis for the MD trajectory of wild type model and comparison of selected QM/MM model and the most representative structure in the dominant cluster (Cluster 1). The overall model structures were shown in ribbon form and key residues in catalytic site were displayed with ball and sticks model. The most representative structure in Cluster 1 was labeled in cyan and the selected QM/MM model was labeled in green. Some crucial distances in representative structure (outside of bracket) and QM/MM model (inside of bracket) were shown with unit of Å.


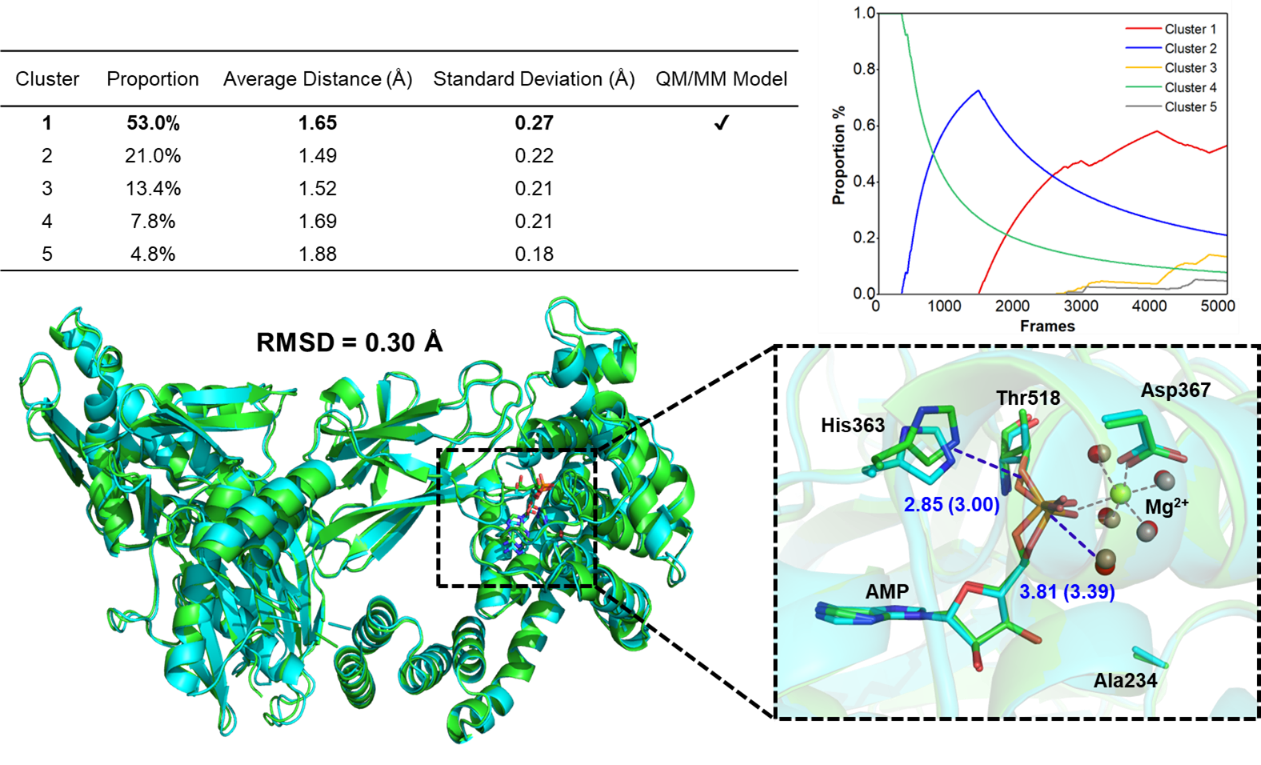


**Figure S3.** Cluster analysis for the MD trajectory of Glu234Ala mutant model and comparison of selected QM/MM model and the most representative structure in the dominant cluster (Cluster 1). The detailed figure legend is the same with Figure S2.

**Figure S4.** The root-mean-square deviations (RMSD) during MD simulations for wild type system **(a)** and Glu234Ala mutant system **(b)**.

**Figure S5.** Variations of dihedral angle of O_α1-_O_α2-_O_α3-_P_α_ along the deAMPylation process in wild type system **(a)** and Glu234Ala mutant system **(b)**.

**Figure S6.** Variations of crucial distances along the deAMPylation process with wild type **(a)** and Glu234Ala mutant system **(b)**.

**Figure S7.** Angle distribution of O_δ4_-P_α_-O_w_ along the deAMPylation process in wild type system **(a)** and Glu234Ala mutant system **(b)**.


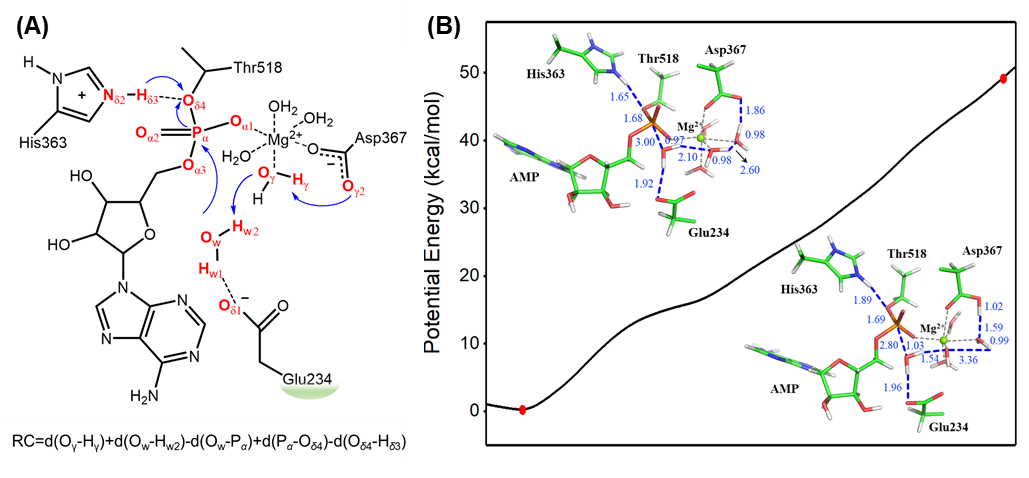


**Figure S8.** **(A)** Schematic research model and defined reaction coordinates for the alternative deAMPylation mechanism in wild type. The electron transfer path of deAMPylation is labeled in blue arrows and atoms involved directly in this process are colored in red. **(B)** Potential energy curve of the alternative mechanism and representative structures along it. Atoms are colored for clarity, C (green), P (orange), O (red), N (blue), and H (white). Distances are given in angstrom.

**Table S1**. Crucial information for deAMPylation and AMPylation in FICD. Distances are given in angstrom. r1, r2, r3 and r7 are followed by Mode-l~3 of Figure 1 in the previous AMPylation research (Liu et al., 2021).

|  | Bond | R | TS | P |
| --- | --- | --- | --- | --- |
| deAMPylation  in wild type  (17.3 kcal/mol) | O$\delta1$-H_w_ | 1.92 | 1.66 | 1.01 |
|  | O_w_-H_w_ | 0.99 | 1.03 | 1.70 |
|  | O_w_-P$\alpha$ | 3.00 | 2.05 | 1.69 |
|  | P$\alpha$-O$\delta4$ | 1.68 | 2.06 | 3.08 |
|  | O$\delta4$-H$\delta3$ | 1.65 | 1.01 | 0.99 |
|  | N$\delta2$-H$\delta3$ | 1.06 | 1.81 | 3.76 |
| deAMPylation  in Glu234Ala mutant  (17.1 kcal/mol) | O$\gamma2$-H$\gamma$ | 5.54 | 1.13 | 0.99 |
|  | O$\gamma$-H$\gamma$ | 0.99 | 1.32 | 3.49 |
|  | O$\gamma$ -H_w_ | 3.16 | 1.17 | 0.98 |
|  | O_w_-H_w_ | 0.98 | 1.25 | 1.92 |
|  | O_w_-P$\alpha$ | 3.39 | 1.83 | 1.65 |
|  | P$\alpha$-O$\delta4$ | 1.61 | 2.36 | 2.84 |
|  | O$\delta4$-H$\delta3$ | 2.40 | 1.01 | 0.98 |
|  | N$\delta2$-H$\delta3$ | 1.02 | 1.89 | 2.05 |
| AMPylation  Model-1  (38.7 kcal/mol) | O_1_-H_His363_ (r1) | 1.00 | 1.74 | 2.10 |
|  | N$\delta2$-H$\delta3$(r2) | 1.82 | 1.07 | 1.02 |
|  | P$\alpha$-O$\delta4$(r3) | 4.43 | 2.20 | 1.71 |
|  | P$\alpha$-O$\alpha\beta$(r7) | 1.65 | 2.13 | 3.20 |
| AMPylation  Model-2  (31.1 kcal/mol) | O_1_-H_His363_ (r1) | 1.00 | 1.90 | 2.10 |
|  | N$\delta2$-H$\delta3$(r2) | 1.82 | 1.05 | 1.02 |
|  | P$\alpha$-O$\delta4$(r3) | 4.40 | 2.03 | 1.70 |
|  | P$\alpha$-O$\alpha\beta$(r7) | 1.64 | 1.89 | 2.96 |
| AMPylation  Model-3  (14.7 kcal/mol) | O_1_-H_His363_ (r1) | 0.98 | 1.02 | 1.83 |
|  | N$\delta2$-H$\delta3$(r2) | 1.87 | 1.68 | 1.03 |
|  | P$\alpha$-O$\delta4$(r3) | 3.82 | 2.02 | 1.66 |
|  | P$\alpha$-O$\alpha\beta$(r7) | 1.66 | 2.10 | 3.25 |

**Table S2.** predicted pKa values for titratable residues.

| Protein | Residue Name | Residue Number | pKa-Model | pKa-Predited |
| --- | --- | --- | --- | --- |
| BiP | GLY | 28 | 8 | 7.23 |
| BiP | ASP | 34 | 3.8 | 7.35 |
| BiP | TYR | 39 | 10 | 14.9 |
| BiP | CYS | 41 | 9 | 12.1 |
| BiP | LYS | 46 | 10.5 | 9.59 |
| BiP | ARG | 49 | 12.5 | 11.87 |
| BiP | GLU | 51 | 4.5 | 2.5 |
| BiP | ASP | 56 | 3.8 | 2.94 |
| BiP | ARG | 60 | 12.5 | 12 |
| BiP | TYR | 65 | 10 | 12.9 |
| BiP | GLU | 71 | 4.5 | 3.2 |
| BiP | GLU | 73 | 4.5 | 3.66 |
| BiP | ARG | 74 | 12.5 | 11.5 |
| BiP | ASP | 78 | 3.8 | 2.49 |
| BiP | LYS | 81 | 10.5 | 10.98 |
| BiP | GLU | 89 | 4.5 | 3.02 |
| BiP | LYS | 96 | 10.5 | 9.98 |
| BiP | ARG | 97 | 12.5 | 14.16 |
| BiP | ARG | 101 | 12.5 | 11.35 |
| BiP | ASP | 105 | 3.8 | 1.19 |
| BiP | ASP | 111 | 3.8 | -0.68 |
| BiP | LYS | 113 | 10.5 | 9.87 |
| BiP | LYS | 118 | 10.5 | 10.5 |
| BiP | GLU | 121 | 4.5 | 4.71 |
| BiP | LYS | 122 | 10.5 | 10.08 |
| BiP | LYS | 123 | 10.5 | 9.8 |
| BiP | LYS | 125 | 10.5 | 9.14 |
| BiP | TYR | 127 | 10 | 12.85 |
| BiP | ASP | 131 | 3.8 | 0.97 |
| BiP | LYS | 138 | 10.5 | 12.01 |
| BiP | GLU | 143 | 4.5 | 6.6 |
| BiP | GLU | 144 | 4.5 | 3.1 |
| BiP | LYS | 152 | 10.5 | 11.11 |
| BiP | LYS | 154 | 10.5 | 11.33 |
| BiP | GLU | 155 | 4.5 | 2.37 |
| BiP | GLU | 158 | 4.5 | 3.44 |
| BiP | TYR | 160 | 10 | 10.47 |
| BiP | LYS | 163 | 10.5 | 10.43 |
| BiP | LYS | 164 | 10.5 | 10.5 |
| BiP | HIE | 167 | 6.5 | 6.36 |
| BiP | TYR | 175 | 10 | 11.58 |
| BiP | ASP | 178 | 3.8 | 2.1 |
| BiP | ARG | 181 | 12.5 | 9.05 |
| BiP | LYS | 185 | 10.5 | 10 |
| BiP | ASP | 186 | 3.8 | 4.98 |
| BiP | ARG | 197 | 12.5 | 12.87 |
| BiP | GLU | 201 | 4.5 | 5.5 |
| BiP | TYR | 209 | 10 | 15.56 |
| BiP | ASP | 212 | 3.8 | 2.97 |
| BiP | LYS | 213 | 10.5 | 9.8 |
| BiP | ARG | 214 | 12.5 | 12.29 |
| BiP | GLU | 215 | 4.5 | 4.08 |
| BiP | GLU | 217 | 4.5 | 3.97 |
| BiP | LYS | 218 | 10.5 | 10.36 |
| BiP | ASP | 224 | 3.8 | 10.26 |
| BiP | ASP | 231 | 3.8 | 6.01 |
| BiP | ASP | 238 | 3.8 | -0.46 |
| BiP | GLU | 243 | 4.5 | 1.17 |
| BiP | ASP | 250 | 3.8 | 4.64 |
| BiP | HIE | 252 | 6.5 | -0.85 |
| BiP | GLU | 256 | 4.5 | 4.25 |
| BiP | ASP | 257 | 3.8 | 2.23 |
| BiP | ASP | 259 | 3.8 | 3.05 |
| BiP | ARG | 261 | 12.5 | 11.62 |
| BiP | GLU | 264 | 4.5 | 4.51 |
| BiP | HID | 265 | 6.5 | 4.33 |
| BiP | LYS | 268 | 10.5 | 10.43 |
| BiP | TYR | 270 | 10 | 13.02 |
| BiP | LYS | 271 | 10.5 | 10.5 |
| BiP | LYS | 272 | 10.5 | 10.5 |
| BiP | LYS | 273 | 10.5 | 10.22 |
| BiP | LYS | 276 | 10.5 | 10.29 |
| BiP | ASP | 277 | 3.8 | 2.34 |
| BiP | ARG | 279 | 12.5 | 12.01 |
| BiP | LYS | 280 | 10.5 | 10.5 |
| BiP | ASP | 281 | 3.8 | 2.68 |
| BiP | ARG | 283 | 12.5 | 11.73 |
| BiP | LYS | 287 | 10.5 | 9.87 |
| BiP | ARG | 289 | 12.5 | 12.92 |
| BiP | ARG | 290 | 12.5 | 12.36 |
| BiP | GLU | 291 | 4.5 | 3.98 |
| BiP | GLU | 293 | 4.5 | 2.45 |
| BiP | LYS | 294 | 10.5 | 10.15 |
| BiP | LYS | 296 | 10.5 | 9.74 |
| BiP | ARG | 297 | 12.5 | 12.5 |
| BiP | HIE | 303 | 6.5 | 6.22 |
| BiP | ARG | 306 | 12.5 | 11.31 |
| BiP | GLU | 308 | 4.5 | 3.51 |
| BiP | GLU | 310 | 4.5 | 4.5 |
| BiP | GLU | 314 | 4.5 | 3.98 |
| BiP | GLU | 316 | 4.5 | 3.9 |
| BiP | ASP | 317 | 3.8 | 3.17 |
| BiP | GLU | 320 | 4.5 | 3.36 |
| BiP | ARG | 324 | 12.5 | 11.17 |
| BiP | LYS | 326 | 10.5 | 10.29 |
| BiP | GLU | 328 | 4.5 | 4.74 |
| BiP | GLU | 329 | 4.5 | 3.98 |
| BiP | ASP | 333 | 3.8 | 2.35 |
| BiP | ARG | 336 | 12.5 | 12.01 |
| BiP | LYS | 340 | 10.5 | 10.5 |
| BiP | LYS | 344 | 10.5 | 10.29 |
| BiP | GLU | 347 | 4.5 | 4.64 |
| BiP | ASP | 348 | 3.8 | 2.5 |
| BiP | ASP | 350 | 3.8 | 3.8 |
| BiP | LYS | 352 | 10.5 | 10.36 |
| BiP | LYS | 353 | 10.5 | 10.29 |
| BiP | ASP | 355 | 3.8 | 2.9 |
| BiP | ASP | 357 | 3.8 | 2.82 |
| BiP | GLU | 358 | 4.5 | 4.7 |
| BiP | ARG | 367 | 12.5 | 13.67 |
| BiP | LYS | 370 | 10.5 | 10.15 |
| BiP | LYS | 376 | 10.5 | 10.22 |
| BiP | GLU | 377 | 4.5 | 3.62 |
| BiP | LYS | 382 | 10.5 | 10.01 |
| BiP | GLU | 383 | 4.5 | 3.01 |
| BiP | ARG | 386 | 12.5 | 12.15 |
| BiP | ASP | 391 | 3.8 | 1.88 |
| BiP | GLU | 392 | 4.5 | 3.25 |
| BiP | TYR | 396 | 10 | 10.2 |
| BiP | ASP | 408 | 3.8 | 1.36 |
| BiP | ASP | 410 | 3.8 | 3.8 |
| BiP | ASP | 413 | 3.8 | 3.09 |
| BiP | ASP | 418 | 3.8 | 4.55 |
| BiP | CYS | 420 | 9 | 8.01 |
| BiP | GLU | 427 | 4.5 | 3.39 |
| BiP | LYS | 435 | 10.5 | 9.87 |
| BiP | ARG | 439 | 12.5 | 11.94 |
| BiP | LYS | 446 | 10.5 | 10.97 |
| BiP | LYS | 447 | 10.5 | 10.43 |
| BiP | ASP | 456 | 3.8 | 3.11 |
| BiP | LYS | 464 | 10.5 | 10.29 |
| BiP | TYR | 466 | 10 | 12.68 |
| BiP | GLU | 467 | 4.5 | 3.81 |
| BiP | GLU | 469 | 4.5 | 2.92 |
| BiP | ARG | 470 | 12.5 | 12.15 |
| BiP | LYS | 474 | 10.5 | 10.29 |
| BiP | ASP | 475 | 3.8 | 2.53 |
| BiP | HID | 477 | 6.5 | 7.34 |
| BiP | ASP | 483 | 3.8 | 3.87 |
| BiP | ARG | 492 | 12.5 | 12.36 |
| BiP | GLU | 498 | 4.5 | -1.74 |
| BiP | GLU | 502 | 4.5 | 3.95 |
| BiP | ASP | 504 | 3.8 | 3.31 |
| BiP | ARG | 510 | 12.5 | 12.08 |
| BiP | GLU | 514 | 4.5 | 2.8 |
| BiP | ASP | 515 | 3.8 | 5.14 |
| BiP | LYS | 516 | 10.5 | 15.6 |
| BiP | AMP | 518 | 12 | 10.2 |
| BiP | LYS | 521 | 10.5 | 9.87 |
| BiP | LYS | 523 | 10.5 | 10.5 |
| BiP | ASP | 529 | 3.8 | 3.1 |
| BiP | ARG | 532 | 12.5 | 12.9 |
| BiP | GLU | 536 | 4.5 | 3.74 |
| BiP | GLU | 537 | 4.5 | 2.55 |
| BiP | GLU | 539 | 4.5 | 3.77 |
| BiP | ARG | 540 | 12.5 | 12.22 |
| BiP | ASP | 544 | 3.8 | 2.62 |
| BiP | GLU | 546 | 4.5 | 4.64 |
| BiP | LYS | 547 | 10.5 | 10.36 |
| FICD | SER | 103 | 8 | 7.72 |
| FICD | GLU | 105 | 4.5 | 2.25 |
| FICD | ARG | 107 | 12.5 | 12.01 |
| FICD | GLU | 115 | 4.5 | 2.91 |
| FICD | LYS | 117 | 10.5 | 11.04 |
| FICD | ARG | 118 | 12.5 | 12.08 |
| FICD | LYS | 121 | 10.5 | 9.66 |
| FICD | ARG | 122 | 12.5 | 11.38 |
| FICD | GLU | 123 | 4.5 | 4.5 |
| FICD | LYS | 124 | 10.5 | 13.1 |
| FICD | LYS | 127 | 10.5 | 11.52 |
| FICD | HIE | 131 | 6.5 | 1.42 |
| FICD | LYS | 134 | 10.5 | 9.08 |
| FICD | ASP | 136 | 3.8 | 2.95 |
| FICD | ASP | 138 | 3.8 | 4.08 |
| FICD | ASP | 141 | 3.8 | 2.7 |
| FICD | GLU | 145 | 4.5 | 3.8 |
| FICD | GLU | 151 | 4.5 | 3.11 |
| FICD | GLU | 152 | 4.5 | 2.91 |
| FICD | ASP | 153 | 3.8 | 3.59 |
| FICD | LYS | 154 | 10.5 | 10.43 |
| FICD | ASP | 155 | 3.8 | 3.56 |
| FICD | ASP | 160 | 3.8 | -2.6 |
| FICD | TYR | 161 | 10 | 10.15 |
| FICD | TYR | 163 | 10 | 13.92 |
| FICD | ARG | 165 | 12.5 | 15.96 |
| FICD | TYR | 172 | 10 | 10 |
| FICD | HIE | 173 | 6.5 | 6.58 |
| FICD | GLU | 174 | 4.5 | 4.5 |
| FICD | LYS | 175 | 10.5 | 10.43 |
| FICD | ARG | 180 | 12.5 | 11.89 |
| FICD | ASP | 181 | 3.8 | 3.91 |
| FICD | ARG | 182 | 12.5 | 11.59 |
| FICD | GLU | 188 | 4.5 | 4.64 |
| FICD | GLU | 189 | 4.5 | 4.5 |
| FICD | ASP | 191 | 3.8 | 1.29 |
| FICD | ARG | 193 | 12.5 | 12.08 |
| FICD | TYR | 194 | 10 | 13.42 |
| FICD | ASP | 199 | 3.8 | -0.16 |
| FICD | LYS | 201 | 10.5 | 10.08 |
| FICD | LYS | 203 | 10.5 | 10.5 |
| FICD | LYS | 204 | 10.5 | 10.5 |
| FICD | LYS | 210 | 10.5 | 10.5 |
| FICD | ARG | 216 | 12.5 | 12.36 |
| FICD | ARG | 217 | 12.5 | 12.01 |
| FICD | GLU | 220 | 4.5 | 2.34 |
| FICD | GLU | 221 | 4.5 | 4.28 |
| FICD | TYR | 223 | 10 | 10.43 |
| FICD | TYR | 224 | 10 | 13.89 |
| FICD | HID | 225 | 6.5 | 5.19 |
| FICD | HID | 226 | 6.5 | 2.27 |
| FICD | TYR | 228 | 10 | 11.37 |
| FICD | HIE | 229 | 6.5 | -0.63 |
| FICD | GLU | 234 | 4.5 | -1.33 |
| FICD | GLU | 242 | 4.5 | 2.99 |
| FICD | ARG | 244 | 12.5 | 11.73 |
| FICD | HID | 245 | 6.5 | 6.43 |
| FICD | GLU | 248 | 4.5 | 3.37 |
| FICD | ARG | 250 | 12.5 | 12.22 |
| FICD | TYR | 251 | 10 | 10 |
| FICD | LYS | 256 | 10.5 | 9.73 |
| FICD | ASP | 258 | 3.8 | 3.84 |
| FICD | GLU | 259 | 4.5 | 4.99 |
| FICD | GLU | 260 | 4.5 | 1.74 |
| FICD | GLU | 263 | 4.5 | 5.06 |
| FICD | HIE | 268 | 6.5 | 4.47 |
| FICD | LYS | 272 | 10.5 | 10.22 |
| FICD | TYR | 273 | 10 | 12.84 |
| FICD | ARG | 281 | 12.5 | 11.94 |
| FICD | ASP | 289 | 3.8 | 1.13 |
| FICD | GLU | 292 | 4.5 | 2.96 |
| FICD | HIE | 294 | 6.5 | 2.88 |
| FICD | ARG | 295 | 12.5 | 12.08 |
| FICD | ARG | 296 | 12.5 | 11.31 |
| FICD | TYR | 300 | 10 | 10 |
| FICD | ASP | 302 | 3.8 | 2.73 |
| FICD | GLU | 305 | 4.5 | 4.05 |
| FICD | ARG | 308 | 12.5 | 11.87 |
| FICD | ARG | 310 | 12.5 | 10.79 |
| FICD | HIP | 318 | 6.5 | 9.58 |
| FICD | HIE | 319 | 6.5 | 3.47 |
| FICD | HID | 323 | 6.5 | 7.23 |
| FICD | ASP | 326 | 3.8 | 3.76 |
| FICD | GLU | 328 | 4.5 | 3.91 |
| FICD | LYS | 329 | 10.5 | 10.43 |
| FICD | GLU | 333 | 4.5 | 4.78 |
| FICD | GLU | 341 | 4.5 | 4.5 |
| FICD | GLU | 342 | 4.5 | 3.91 |
| FICD | HIE | 347 | 6.5 | 7.19 |
| FICD | GLU | 350 | 4.5 | 5.29 |
| FICD | HID | 356 | 6.5 | -0.09 |
| FICD | TYR | 357 | 10 | 12.46 |
| FICD | LYS | 358 | 10.5 | 9.85 |
| FICD | TYR | 361 | 10 | 10.12 |
| FICD | HIP | 363 | 6.5 | 0.43 |
| FICD | ASP | 367 | 3.8 | 0.89 |
| FICD | ARG | 371 | 12.5 | 12.99 |
| FICD | ARG | 374 | 12.5 | 12.12 |
| FICD | TYR | 386 | 10 | 13.58 |
| FICD | ARG | 392 | 12.5 | 11.74 |
| FICD | LYS | 393 | 10.5 | 10.29 |
| FICD | GLU | 394 | 4.5 | 3.86 |
| FICD | ARG | 396 | 12.5 | 11.61 |
| FICD | ASP | 398 | 3.8 | 3.8 |
| FICD | TYR | 399 | 10 | 8.62 |
| FICD | TYR | 400 | 10 | 12.63 |
| FICD | HIE | 401 | 6.5 | 5.62 |
| FICD | GLU | 404 | 4.5 | 6.8 |
| FICD | GLU | 408 | 4.5 | 1.02 |
| FICD | ASP | 410 | 3.8 | 1.69 |
| FICD | ARG | 412 | 12.5 | 15.42 |
| FICD | ARG | 416 | 12.5 | 11.87 |
| FICD | LYS | 420 | 10.5 | 10.36 |
| FICD | CYS | 421 | 9 | 9.46 |
| FICD | GLU | 423 | 4.5 | 3.03 |
| FICD | ASP | 427 | 3.8 | 3.78 |
| FICD | GLU | 435 | 4.5 | 4.65 |
| FICD | TYR | 436 | 10 | 10 |
| FICD | GLU | 442 | 3.2 | 3.34 |
| FICD | GLU | 442 | 4.5 | 4.57 |
| FICD | HIE | 131 | 13.5 | 9.73 |
| FICD | HIE | 173 | 13.5 | 13.58 |
| FICD | HID | 225 | 13.5 | 12.31 |
| FICD | HID | 226 | 13.5 | 9.27 |
| FICD | HIE | 229 | 13.5 | 8.21 |
| FICD | HID | 245 | 13.5 | 13.43 |
| FICD | HIE | 268 | 13.5 | 13.87 |
| FICD | HIE | 294 | 13.5 | 9.88 |
| FICD | HIP | 318 | 13.5 | 17.07 |
| FICD | HIE | 319 | 13.5 | 12.24 |
| FICD | HID | 323 | 13.5 | 14.23 |
| FICD | HIE | 347 | 13.5 | 16.32 |
| FICD | HID | 356 | 13.5 | 9.31 |
| FICD | HIP | 363 | 13.5 | 8.23 |
| FICD | HIE | 401 | 13.5 | 12.65 |

**References**

Liu, M.; Huai, Z.; Tan, H.; Chen, G. (2021) Investigation of the Detailed AMPylated Reaction Mechanism for the Huntingtin Yeast-Interacting Protein E Enzyme HYPE. *Int J Mol Sci*, *22* (13). doi: 10.3390/ijms22136999.
